# Quality and Guideline Adherence of Mobile Nutrition Management Apps for Diabetes: Evaluation Study

**DOI:** 10.2196/80890

**Published:** 2026-06-01

**Authors:** Sidra Jahanzeb, Nadia Davoody

**Affiliations:** 1 Health Informatics Centre, Department of Learning, Informatics, Management and Ethics, Karolinska Institutet, Tomtebodavägen 18 A, Stockholm, Stockholm County, S-17177, Sweden, 46 0 8 524 864

**Keywords:** diabetes mellitus, mobile apps, dietary guidelines, self-management, MARS, mHealth apps, evaluation, Mobile App Rating Scale, mobile health

## Abstract

**Background:**

Diabetes mellitus is a chronic metabolic disorder marked by elevated blood glucose levels and has emerged as a global epidemic that requires management strategies for effective glycemic control through diet. In recent years, mobile apps have emerged as valuable tools for supporting self-management in chronic diseases such as diabetes, particularly for the nutritional aspects of the disease. However, the quality, accuracy, and adherence of these apps to established dietary guidelines remain underexplored and inconsistent.

**Objective:**

The study aims to evaluate the quality and adherence to guidelines of digital nutrition management apps for diabetes, with a focus on dietary guidelines from the American Diabetes Association (ADA), World Health Organization (WHO), European Association for the Study of Diabetes (EASD), and Diabetes Canada (DC).

**Methods:**

A quality evaluation was performed, involving the identification of mobile apps from the Google Play Store and Apple App Store. A total of 24 apps were selected based on predefined inclusion and exclusion criteria. Apps were analyzed for their content and features using a compliance checklist derived from official dietary guidelines for diabetes, including carbohydrate tracking, meal planning, glycemic control, fiber intake, physical activity, weight management, and education about diabetes. Additionally, the Mobile App Rating Scale was used to evaluate app quality in terms of engagement, functionality, aesthetics, and information. Intraclass correlation coefficients were used to calculate interrater reliability. Pearson correlation coefficients were also calculated.

**Results:**

Only 2 apps showed full compliance with the dietary guidelines, while most apps showed partial adherence. The Mobile App Rating Scale evaluation revealed significant variability in app quality, with mean total scores ranging from 9.46 to 17.69. This finding indicated major gaps in user engagement, functionality, educational content, and personalization. The intraclass correlation coefficient was 0.86, which indicates good interrater reliability, and the Pearson correlation coefficient was 0.88, suggesting good consistency between the authors.

**Conclusions:**

Even with the growing availability of nutrition management apps for diabetes, many lack full compliance with dietary guidelines and show room for improvement in their content quality. Collaboration between health care professionals, developers, and patients is essential for the future development of these tools to effectively support diabetes self-management. Strengthening guideline adherence and content quality can increase the effectiveness of these digital tools in promoting self-management and improving health outcomes for individuals with diabetes.

## Introduction

### Diabetes Mellitus

Diabetes mellitus is a chronic metabolic disorder marked by elevated blood glucose levels and has emerged as a global epidemic. According to the World Health Organization (WHO), “Diabetes is a chronic, metabolic disease characterized by elevated levels of blood glucose (or blood sugar), which leads over time to serious damage to the heart, blood vessels, eyes, kidneys, and nerves” [1].

It is one of the leading causes of morbidity and mortality, and it is often called the “silent killer” due to its slow onset and the absence of obvious symptoms in its early stages, which can lead to severe long-term complications if left undiagnosed and untreated, including cardiovascular disease, kidney failure, neuropathy, and retinopathy. These complications significantly affect the quality of life of individuals and lead to increased health care costs [2,3].

### Role of Diet in Diabetes Management

Diabetes management is effective through combining medical treatment, lifestyle modification, and dietary changes. Dietary management is one of the most important aspects of diabetes care and emphasizes carbohydrate counting, prioritizing foods with a low glycemic index, intake of high-fiber foods, and a balanced diet of macronutrients [4,5].

Dietary guidelines for diabetes management have been developed by the American Diabetes Association (ADA), WHO, the European Association for the Study of Diabetes (EASD), and the Canadian Diabetes Association (CDA). These guidelines are recognized worldwide by health care professionals and play an important role in effective diabetes management [6]. Despite the availability of these guidelines, many individuals still struggle with dietary adherence because of a lack of education and little to no access to professional support [7].

Although the main goal of dietary management in diabetes is blood glucose control, its benefits extend beyond glycemic management. A balanced and healthy diet can help patients with diabetes manage other risk factors, such as hypertension and high cholesterol levels, which are common among people with diabetes [8].

### Digital Health Solutions and Their Challenges for Nutrition Management of Diabetes

In recent years, digital tools for health care, particularly mobile health (mHealth) apps for self-managing diseases, have taken the industry by storm. These mobile apps have many functionalities, including nutritional information, meal planning, carbohydrate counting, monitoring physical activity, and medication reminders, among others [7]. With the help of these mHealth apps, patients can easily access their nutritional data, track their progress, and receive reminder notifications about their activities, which in turn may help patients remain mindful of their eating habits and other activities and improve their ability to manage their condition [9,10].

mHealth apps may have the potential to act as a bridge between patients and health care providers by providing personalized recommendations. These apps can also be a source of educational content that can help patients understand the importance of dietary choices and their effect on blood glucose levels [11]. Moreover, some apps also include a feature of barcode scanning for food products that allows users to access nutritional information about whether the product is compatible with their dietary needs. Some apps are integrated with wearable devices (eg, fitness trackers and glucose monitors) to give real-time glucose levels and feedback that further increase the ability of the user in managing their condition [12].

The use of digital tools in diabetes management is not limited to mHealth apps. Other technologies have also come into play, such as continuous glucose monitoring systems and insulin pumps, which have also been integrated with mHealth apps [13]. However, there are concerns regarding the quality, credibility, and adherence of these mHealth apps to established dietary guidelines [14].

To support diabetes management, these apps need to have a solid background that supports and aligns with diabetic nutrition guidelines, which many apps fail to do. Without this, these apps may not be able to assist patients in managing their condition, which can lead to undesirable and potentially dangerous health outcomes [15]. Therefore, a systematic evaluation of these apps in relation to official dietary guidelines is necessary to assess their functionality and identify areas for improvement.

### Aim and Objective of the Study

This study aimed to evaluate the quality and adherence to guidelines of digital nutrition management apps for diabetes, with a focus on the main dietary guidelines from the ADA, WHO, EASD, and Diabetes Canada (DC). This study compared the features of various apps, assessed whether they comply with dietary guidelines, and provided recommendations for improvement.

## Methods

### Study Design and Setting

**Figure 1.**
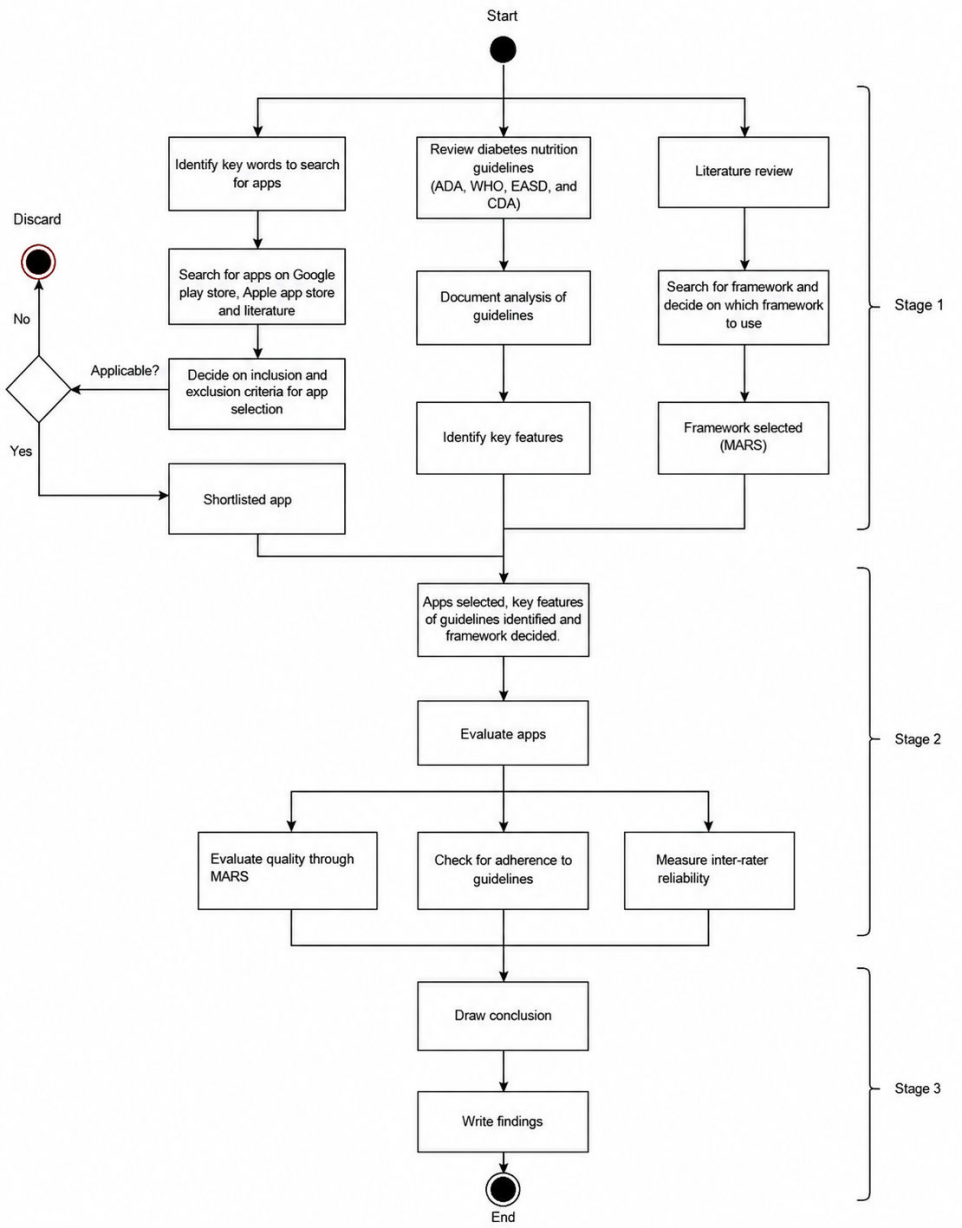
Unified Modeling Language (UML) activity diagram illustrating phases of the study. ADA: American Diabetes Association; CDA: Canadian Diabetes Association; EASD: European Association for the Study of Diabetes; MARS: Mobile App Rating Scale; WHO: World Health Organization.

This section provides an overview of the research methodology used in this study. It consisted of several stages where work was divided into several sections, and some of these sections took place simultaneously. A Unified Modeling Language diagram in Figure 1 shows all the phases used throughout this study.

This evaluation study assessed the quality and guideline compliance of mHealth apps aimed at supporting nutrition management for individuals with diabetes. The research was conducted in 3 stages that involved multiple steps in each stage to achieve the desired result. The research approach was designed to ensure a thorough selection process such that no relevant app was missed, and the selected mHealth apps were functionally adequate and met the study criteria.

The study was conducted in 2025, with data collection and analysis carried out between January 17, 2025, and June 11, 2025. The primary source of app selection was the Google Play Store, Apple App Store, and existing literature, from which mHealth apps were selected based on predefined inclusion and exclusion criteria. The analysis was conducted on publicly available apps, dietary guidelines, and evaluation frameworks, with all research taking place in Stockholm, Sweden.

### Data Collection

A systematic search strategy was used to identify eligible apps. The app identification process was performed in two stages: searching the Apple App Store and Google Play Store and searching the literature to identify additional apps reported in previous studies [11,12,15]. The databases used for the literature search were PubMed, Web of Science, and CINAHL. The search strategy used keywords related to diabetes, mobile apps, and nutrition management. The following search terms were used for each database: (“diabetes” OR “diabetes mellitus” OR “type 1 diabetes” OR “type 2 diabetes”) AND (“mobile app” OR “mobile application” OR “mHealth” OR “smartphone app”) AND (“nutrition” OR “diet” OR “meal planning” OR “carbohydrate counting” OR “food tracking” OR “diet management”)

The search was performed on English-language publications only. Titles and abstracts were screened to identify relevant studies evaluating or mentioning mobile apps for diabetes nutrition management.

Similarly, the Google Play Store and Apple App Store were searched systematically for apps using predefined keywords. The search was performed in February 2025. The following keywords were used in both app stores: “diabetes,” “nutrition management,” “meal planning,” “diabetes management,” “diabetes diet,” “carb counting,” “diabetes meal tracking,” “glucose control,” “glycemic index,” “diabetes recipes,” “diabetes food tracker,” and “diabetes nutrition app.”

For apps downloaded from the Google Play Store, a Samsung S22 Ultra with Android (version 14) was used, and an iPhone 14 Pro Max with iOS (version 18.3.2) was used to download apps from the Apple App Store. The search was performed through a systematic search, with the same keywords used in the same sequence on both platforms to avoid missing any relevant apps and to ensure consistency. Apps that appeared as similar apps on the app page were also assessed. They were then reviewed through their titles, descriptions, and lists of features to gauge their relevance to the topic. After the initial search, a set of inclusion and exclusion criteria was used to filter the most relevant apps for evaluation. The inclusion and exclusion criteria are shown in Table 1.

**Table 1.** Inclusion and exclusion criteria for app selection.

Criterion	Inclusion criteria	Exclusion criteria
Focus	Apps that focus on diabetes management and nutrition.	Apps that only focus on weight loss without any diabetes or nutrition management.
Availability	Apps are available on Google Play Store and the Apple App Store.	App available on other search engines or requiring institutional access.
Language	Apps available in English.	Apps with insufficient English content are preventing evaluation.
Update	Apps that have been updated in the last 12 months.	Outdated apps and apps that crashed, failed to load, or were not functional during testing.
Category	Apps in the categories of Medical, Health and Fitness, Health Monitoring, Meal Planning, and Education.	Apps in any other category are not included in the inclusion criteria.
Payment	Free to download or apps with some in-app purchases.	Apps requiring a mandatory paid subscription to access core features needed for evaluation.
Target users	Apps intended for individuals with diabetes or general users managing diabetes.	Apps designed to be used by health care professionals only.
Others	—^a^	Apps that require a monetary subscription and cannot be accessed without it.
Others	—^a^	Apps that require external devices to work (eg, glucometer).
Others	—^a^	Duplicates from the Apple App Store and Google Play Store.

a Not available.

The selected apps must have been updated within the last 12 months to focus on actively maintained apps. This criterion ensured that included apps were compatible with current operating systems, received ongoing security and bug fixes, and contained up-to-date content and features. User ratings were not considered as part of the inclusion or exclusion criteria for this study due to potential bias in the ratings. User ratings are often hard to judge due to factors such as individual user experience, initial impressions, and limited knowledge about the app, rather than overall quality, content, or functionality. Moreover, user ratings or reviews may not provide an accurate picture of app quality and functionality, as most users focus more on the usability and interface design rather than the validity or accuracy of the content [16].

All identified apps were screened through a multistage process to ensure relevance and quality. Duplicates were removed, followed by exclusion of apps that did not meet predefined inclusion criteria such as focus on diabetes nutrition, language, update, and accessibility. The screening process was conducted consistently using the same search terms and criteria on both the Apple App Store and Google Play Store.

### Data Analysis

The study was conducted by 2 authors (SJ and ND), both of whom have experience in eHealth design, digital self-management support systems, and evaluation of health technologies using established assessment frameworks. Before conducting the app evaluations, the authors reviewed the methodological literature on the use of the Mobile App Rating Scale (MARS) across various health domains and familiarized themselves thoroughly with its structure, scoring procedures, and appropriate use within app evaluation studies. SJ and ND collaboratively developed the study concept and design. SJ was responsible for the initial data collection, which included searching the literature and app stores and compiling the list of eligible apps. Both authors collaboratively developed a compliance checklist based on dietary guidelines to evaluate features within each app. Both authors then evaluated the included apps for compliance with dietary guidelines. In addition, each author independently assessed app quality using MARS. Interrater reliability was statistically calculated to assess the agreement between the 2 authors, and the results were subsequently reviewed and refined by both authors. The data analysis in this study was performed in 2 parts. The first part involved a review of dietary guidelines from the ADA, WHO, EASD, and DC to identify key dietary recommendations for diabetes management [1,9,17,18]. Although these guidelines differ slightly in how dietary recommendations are presented, the main elements for diabetes management remain the same. These organizations are internationally recognized and provide a gold standard in nutrition, lifestyle, and blood glucose management for diabetes treatment. These guidelines were screened for common nutrition-related recommendations for diabetes, including carbohydrate monitoring, glycemic index guidance, meal planning support, portion control, fiber intake, regular physical activity, and education to support self-management. Both authors independently extracted nutrition-related recommendations from each guideline. Extracted recommendations were then compared across guidelines, and overlapping or similar recommendations were grouped together. Through discussion, these grouped recommendations were translated into checklist items representing dietary features based on the guidelines. The content of mHealth apps was evaluated to determine whether each app complies with the guidelines. Each app was systematically assessed based on whether it included features such as carbohydrate tracking, glycemic index monitoring, meal planning, and other key components of diabetes management, as specified in the guidelines. The final checklist included the following items: carbohydrate control, meal planning, glycemic control, fiber intake, physical activity and exercise, personalized nutrition plans, and education about the disease. The apps were individually evaluated, and their adherence to each guideline was assessed by examining whether they included features corresponding to the features on the compliance checklist and whether these features were implemented appropriately. Apps that met these criteria were classified as compliant, while those lacking the necessary features were labeled as noncompliant.

The second part included evaluating the quality of the selected apps using MARS, which is a widely used tool developed by Stoyan et al [19]. It was chosen due to its defined evaluation framework, which assesses mobile apps in a structured way across different categories and is essential for understanding both content quality and user experience. A 5-point Likert scale is used, where 1 indicates inadequate and 5 indicates excellent. The scale has a total of 29 questions covering app quality, subjective quality, and app-specific ratings. A total of 23 questions were used for the analysis of engagement, functionality, aesthetics, information, and subjective quality, while 6 customizable questions were used to assess app-specific quality. Only the app’s overall quality section was used for this study, which included four categories: engagement, functionality, aesthetics, and information. The selected 4 categories are provided in Textbox 1. App subjective quality was excluded because it requires user feedback, which is outside the scope of this study, and app-specific questions were also excluded. The MARS ratings were determined exclusively based on features available for free within the apps, without considering paid or premium content. Each app was individually assessed by both authors, and the average score across the 4 categories was calculated, with scores ranging from 1 to 5. Therefore, the total score was calculated by dividing the total score across all 4 categories by 4. App developers, health care professionals, and researchers are the primary users of this scale [19], and it has been consistently used in previous studies [20] to evaluate mHealth apps either alone or in combination with other tools, which supports its reliability for this type of analysis.

Textbox 1. **App quality rating aspects assessed using the Mobile Application Rating Scale (MARS).**

**Engagement:**

- Entertainment
- Interest
- Customization
- Interactivity
- Target group

**Functionality:**

- Performance
- Ease of use
- Navigation
- Gestural design

**Aesthetics:**

- Layout
- Graphics
- Visual appeal

**Information quality:**

- Accuracy of app description
- Goals
- Quality of information of app content
- Quality of information regarding the extent of coverage
- Visual information
- Credibility
- Evidence-based

To evaluate interrater reliability for total MARS scores, a 2-way mixed-effects ANOVA was conducted. This is an efficient statistical method available that partitions total variability in the data into separate components to determine how much variation is explained by different sources [21]. The intraclass correlation coefficient (ICC [3,1]) was derived from ANOVA mean squares and estimated absolute agreement between fixed raters. Pearson correlation between the total scores of both authors was also calculated.

The underlying ANOVA model used was as follows:


$$Y_{ij}=\mu +\alpha_{i}+\beta_{j}+\varepsilon_{ij}$$

where Y_ij_ is the MARS score for app *i* by rater *j*, μ is the overall mean, α_i_ is the random effect of app *i*, β_j_ is the fixed effect of rater *j*, and ε_ij_ is the residual error.

The ICC was calculated using the following formula for a 2-way mixed-effects absolute-agreement model with single measures:


$$ICC(3,1)=\frac{MSR-MSE}{MSR+(k-1)MSE}$$

where MRS is the mean square for apps, MSE is the mean square error, and *k* is the number of evaluators, which were 2 in this study. Values below 0.5 indicate poor reliability, 0.5‐0.75 indicate moderate reliability, 0.75‐0.9 indicate good reliability, and values above 0.9 indicate excellent reliability.

### Ethical Considerations

The study adhered to ethical guidelines to ensure that the research was conducted responsibly. Direct human participation and personal data collection were not required because the study focused on analyzing mHealth apps for diabetes management. All selected apps were freely available on the Google Play Store and Apple App Store, and the evaluation was based on publicly available content such as app descriptions, features, and functionalities. As no personal or sensitive data from users were involved, data privacy concerns were minimal. Furthermore, the study used established dietary guidelines that were publicly available from ADA, WHO, EASD, and DC. The study clearly outlined the data sources and provided a transparent description of the methods and procedures to ensure that the research findings can be reliably reproduced. In terms of data management, all collected information was securely stored on password-protected devices, and no personal or identifiable information was recorded at any stage of this research.

## Results

### Overview

This section presents the findings of the evaluation of 24 selected mHealth apps for diabetes nutrition management. The following subsections include results for app screening, description of the apps, evaluation of compliance with dietary guidelines, and MARS evaluation.

### Apps Screening

**Figure 2.**
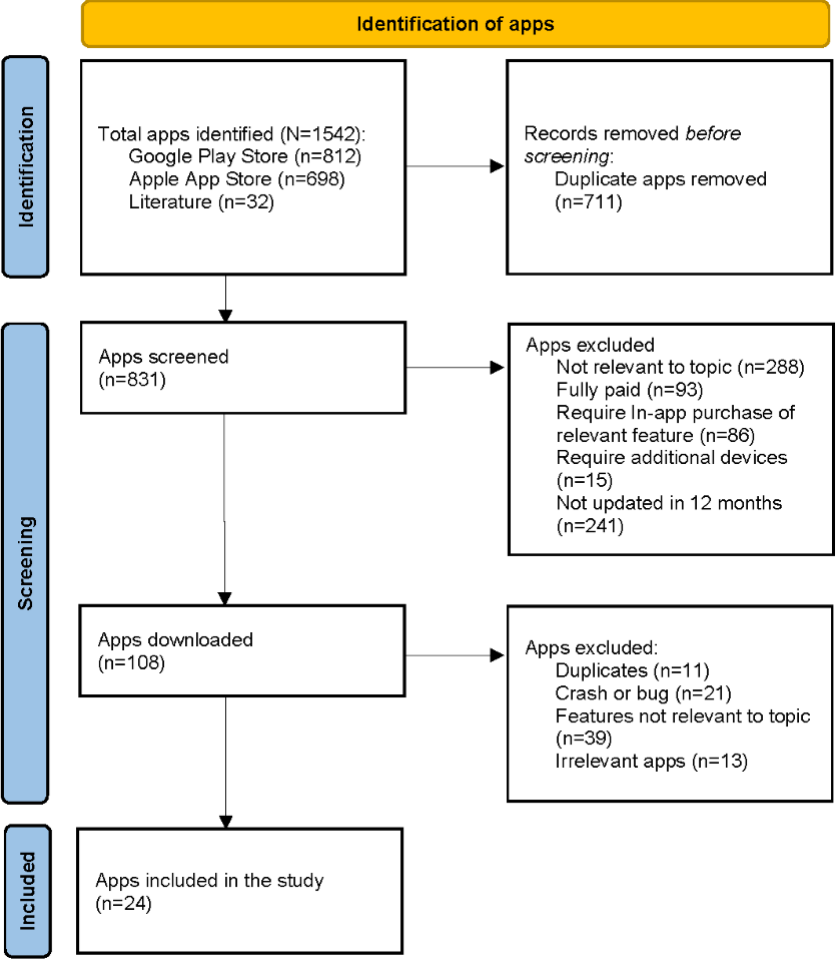
PRISMA (Preferred Reporting Items for Systematic Reviews and Meta-Analyses) flowchart for app identification and screening.

A total of 1542 mobile apps were identified, 812 from Google Play Store, 698 from Apple App Store, and 32 from previous literature. After removing 711 duplicates, 831 apps were screened for eligibility. A total of 108 apps were downloaded for further review after following the inclusion and exclusion criteria. Moreover, 84 apps were excluded due to duplication, crashes, or lack of relevant features, leaving 24 apps for the final evaluation. The PRISMA (Preferred Reporting Items for Systematic Reviews and Meta-Analyses) flowchart for app identification and screening is summarized in Figure 2.

### Apps Features

A basic description of the selected 24 apps used in this study is shown in Table 2. The mHealth apps were selected based on the inclusion and exclusion criteria provided in Table 1. The 24 apps were downloaded from Google Play Store and Apple App Store under the categories of Health and Fitness, Medical, Health Monitoring, Meal Planning, and Food and Drink. All apps were available on both the Apple App Store and the Google Play Store. The categories reflect the main focus of each app, where Health and Fitness apps focus on general wellness, lifestyle modification, diet tracking, and physical activity. Meal Planning apps provide structured meal plans, dietary recommendations, and nutrition guidance. Medical apps are used for disease management and include clinical information, education, and treatment-related support. Health Monitoring apps focus on tracking health parameters such as blood glucose, dietary intake, and other self-management indicators. The Food and Drink category focuses on apps that mainly provide food databases, recipe collections, nutrition information, and meal-tracking features.

**Table 2.** Apps descriptions.

App name	App store category	Developer name	Last update	Cost
Carb Manager	Health, Fitness, and Meal Planning	Wombat Apps LLC	Feb 2025	Free but offers in-app purchases
MyDiabetes: Meal, Carb Tracker	Health, Fitness, and Medical and Health Monitoring	GO Health Solutions	Mar 2025	Free but offers in-app purchases
Yazio	Health and Fitness	YAZIO	Apr 2025	Free but offers in-app purchases
Glycemic Index. Diabetes Diary	Medical	App Holdings	Dec 2024	Free but offers in-app purchases
Health2Sync–Glucose Tracker	Medical and Health Monitoring	H2 Inc	Apr 2025	Free but offers in-app purchases
MyNetDiary	Health and Fitness	MyNetDiary.com	Apr 2025	Free but offers in-app purchases
mySugr	Medical and Health Monitoring	mySugr GmbH	Apr 2025	Free but offers in-app purchases
Glycemic Index & Load App	Health and Fitness	RFIT Development	Mar 2025	Free but offers in-app purchases
Glooko	Health, Fitness, and Medical and Health Monitoring	Glooko Inc	Mar 2025	Free
Carbs & Cals: Diet & Diabetes	Health and Fitness	Chello Publishing Limited	Apr 2025	Free but offers in-app purchases
Center Health	Medical	Center Health Inc	Apr 2025	Free
BeatO	Health, Fitness, and Medical	Health Arx Technologies Private Limited	Feb 2025	Free
DiabTrend	Health, Fitness, and Medical and Health Monitoring	DiabTrend AI Analytics Inc	Jan 2025	Free but offers in-app purchases
Diabetic Diary–Glucose Tracker	Health, Fitness, and Medical	mEL Studio	Feb 2025	Free but offers in-app purchases
Glucose Buddy	Medical and Health Monitoring	Azumio Inc	Jul 2024	Free but offers in-app purchases
Diabetes Recipes Diabetic Diet	Health and Fitness	Edutainment Ventures	Apr 2025	Free but offers in-app purchases
Fooducate	Health and Fitness	Maple Media	Nov 2024	Free but offers in-app purchases
Diabetes:M	Medical	Sirma Medical Systems	Feb 2025	Free but offers in-app purchases
Diabetes Tracker	Medical	Axel Stein	Mar 2025	Free but offers in-app purchases
SocialDiabetes	Medical and Health Monitoring	SocialDiabetes	Apr 2025	Free but offers in-app purchases
Gluroo: Diabetes Log Tracker	Health, Fitness, and Medical and Health Monitoring	Gluroo Imaginations Inc	Apr 2025	Free
nBuddy Diabetes	Health and Fitness	HeartVoice	May 2025	Free but offers in-app purchases
Blood Sugar Diary for Diabetes	Medical and Health Monitoring	MedM Inc	Apr 2025	Free but offers in-app purchases
Diabetes App–Diabetic Diet	Health, Fitness, and Food and Drink	Aquila Soft	Aug 2024	Free but offers in-app purchases

**Figure 3.**
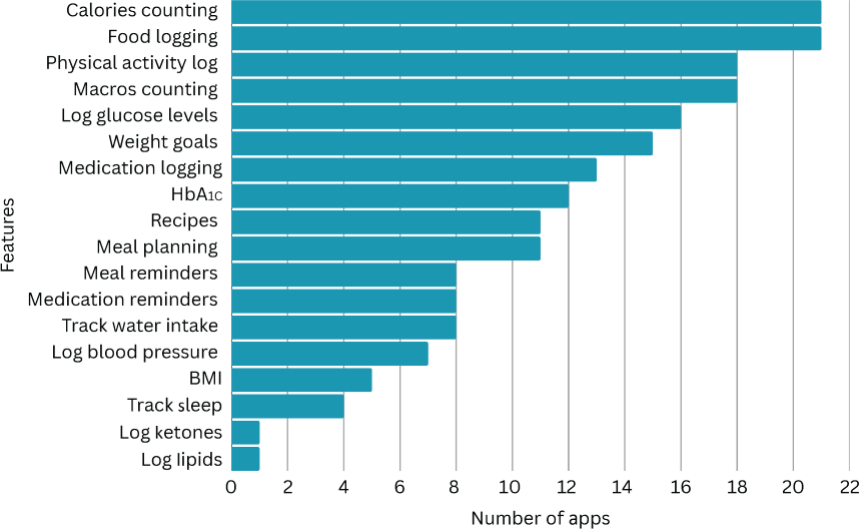
Features corresponding to the number of apps. HbA_1c_: hemoglobin A_1c_.

A detailed overview of the features of all the apps is provided in Multimedia Appendix 1. A bar chart illustrating various features of mHealth apps designed for nutritional management of diabetes, showing the tools users can use to monitor and manage their condition, is provided in Figure 3. The most common features were calorie counting and food logging, each present in 21 of the 24 apps, indicating that most apps prioritized food tracking and calorie counting for diabetes management. Macros counting and physical activity logging were present in 18 apps. The feature to log glucose levels was found in 16 apps, while other features include weight goals (15 apps), medication logging (13 apps), and hemoglobin A_1c_ (HbA_1c_) logging or estimation (12 apps). Less frequently included features, such as water intake tracking (8 apps), blood pressure logging (7 apps), and BMI tracking (5 apps), provided a more comprehensive approach to managing diabetes by addressing other aspects of health, such as hydration and cardiovascular function. The least common features, including ketone logging and lipid logging, were present in only 1 app each.

### Compliance With Dietary Guidelines

The compliance of the selected 24 mHealth apps with official dietary guidelines for diabetes management was evaluated using a checklist formed by reviewing the guidelines to identify the main dietary recommendations and systematically assessing the presence or absence of these features in the apps. The analysis of the selected apps showed varying levels of compliance with essential features, with some offering proper support for diabetes management, while others displayed significant gaps.

**Figure 4.**
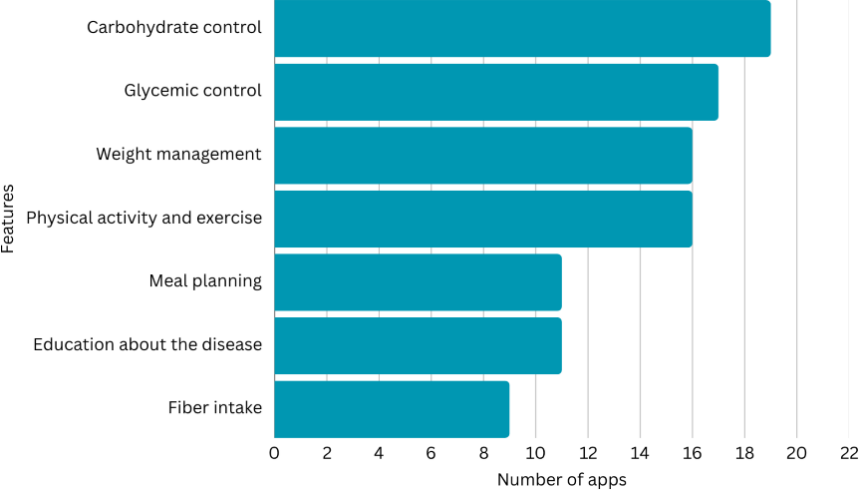
Compliance checklist in relation to the number of apps.

Figure 4 illustrates the number of apps in relation to their compliance features. A detailed overview is provided in Multimedia Appendix 2, where “x” marks compliance with the dietary guidelines.

Only 2 apps, BeatO (Health Arx Technologies Private Limited) and Glycemic Index & Load App (RFIT Development)*,* showed full compliance with dietary guidelines by including features for carbohydrate control, meal planning, glycemic control, fiber intake, physical activity, weight management, and education.

Three apps, including MyDiabetes: Meal, Carb Tracker (GO Health Solutions), Glucose Buddy (Azumio Inc), and Center Health (Center Health Inc), showed strong adherence to dietary guidelines. All these apps met 6 of the 7 checklist features that were in accordance with the dietary guidelines. For instance, MyDiabetes: Meal, Carb Tracker, and Center Health lacked only fiber intake tracking while offering all other features. Similarly, Glucose Buddy lacked only the meal-planning feature.

Five apps, including Health2Sync–Glucose Tracker (H2 Inc), Yazio (YAZIO), SocialDiabetes, DiabTrend (DiabTrend AI Analytics Inc), and nBuddy Diabetes (HeartVoice), each showed compliance with 5 of the 7 key features of the compliance checklist for diabetes management. Health2Sync–Glucose Tracker and SocialDiabetes were compliant with carbohydrate control, glycemic control, physical activity, weight management, and education about the condition, but lacked both meal planning and fiber intake tracking. The app Yazio offered strong support for carbohydrate control, meal planning, physical activity, weight management, and fiber intake, but lacked glycemic control because it did not allow logging of glucose levels and lacked education about the condition. DiabTrend and nBuddy Diabetes were both compliant with carbohydrate control, glycemic control, physical activity, fiber intake, and education about the condition, but lacked both meal planning and weight management features.

Moreover, the apps Carb Manager (Wombat Apps LLC), Gluroo (Imaginations Inc), Glooko (Glooko Inc), Carbs & Cals: Diet & Diabetes (Chello Publishing Limited), and Diabetes Recipes Diabetic Diet (Edutainment Ventures) were compliant with 4 of the 7 key features in the compliance checklist for diabetes management. All these apps provided carbohydrate control. Meal planning was present in Carb Manager, Carbs & Cals: Diet & Diabetes, and Diabetes Recipes Diabetic Diet, whereas the glycemic control feature was present in Gluroo: Diabetes Log Tracker (Gluroo Imaginations Inc) and Glooko. Furthermore, Carb Manager, Gluroo: Diabetes Log Tracker, and Carbs & Cals: Diet & Diabetes encouraged users to increase fiber intake. Weight management was offered only by Carb Manager, Glooko, and Carbs & Cals: Diet & Diabetes, while Gluroo: Diabetes Log Tracker, Glooko, and Diabetes Recipes Diabetic Diet included features to log physical activity and exercise. Finally, only Diabetes Recipes Diabetic Diet provided users with education regarding diabetes. Some apps showed partial compliance. For example, the app mySugr (mySugr GmbH) included only carbohydrate control, glycemic control, and physical activity. Similarly, Diabetes Tracker (Axel Stein), Diabetic Diary–Glucose Tracker (mEL Studio), and Glycemic Index*.* Diabetes Diary (App Holdings) offered features for carbohydrate control through macronutrient counting, glycemic control through tracking and logging blood glucose levels, and weight management through calorie counting and weight-goal assignment.

Additionally, MyNetDiary (MyNetDiary) and Fooducate (Maple Media) offered a unique approach to diabetes management by initially asking users whether they had diabetes or another health condition, which allowed the apps to tailor plans accordingly. These apps focused on meal planning, weight management, and physical activity. Furthermore, the apps Diabetes:M - Blood Sugar Diary (Diabetes Diet) and Diabetes App*–*Diabetes Diet (Aquila Soft) offered only one feature: glycemic control through logging and tracking blood glucose levels.

### MARS Evaluation

The MARS was used to assess the quality of the selected 24 mHealth apps for nutritional management of diabetes. The questions used to perform the MARS evaluation are provided in Multimedia Appendix 3.

The evaluation showed significant variation in the performance of the apps. Two (8%) apps achieved high performance, with a mean total score of 17.76 for Carb Manager and 17.12 for MyDiabetes: Meal, Carb Tracker across all domains.

A larger proportion of the apps showed average performance, amounting to 67% (n=16) of the total apps, with MARS scores ranging from 12 to 16. Some apps performed reasonably well, including Yazio (15.83), Glycemic Index. Diabetes Diary (15.79), Health2Sync–Glucose Tracker (14.41), MyNetDiary (15.23), mySugr (13.75), and Glycemic Index & Load App (14.82).

Six (25%) apps showed low performance, with scores below 12. For example, the Diabetes App–Diabetic Diet scored the lowest among all apps, with a score of 9.46, followed by Blood Sugar Diary for Diabetes (9.9; MedM Inc), Gluroo: Diabetes Log Tracker (10.82), SocialDiabetes (11.49), and Diabetes Tracker (10.94)*.*

**Figure 5.**
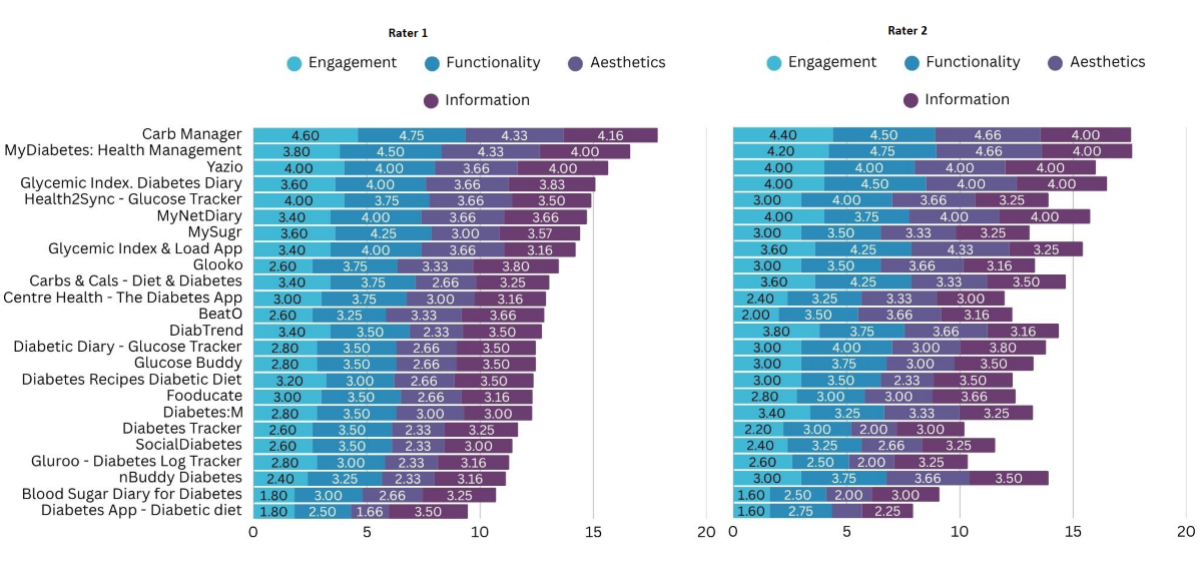
Mobile App Rating Scale (MARS) scores.

Figure 5 shows a stacked bar chart presenting MARS scores from both authors for all apps. A detailed overview of MARS scores, including total and mean total scores for each app, is provided in Multimedia Appendix 4.

A 2-way mixed-effects ANOVA model was performed to assess interrater reliability for the total MARS scores, in which apps were treated as random effects and raters as fixed effects. Through this model, the scores from each app and rater were compiled into three sources of variance: variance between apps, variance between raters, and residual error. The ANOVA partitioned the total variability into these components and generated mean square values. The mean square for rows represented the differences in total MARS scores between the 24 apps, the mean square for columns represented differences between the 2 raters, and MSE represented random disagreement between raters for each app. The calculated mean square values were mean square for rows=18.68, mean square for columns=0.08, and MSE=1.08, and *k*=2 represented the number of raters. Using these values, the ICC (3,1) was calculated through the equation:


$$ICC\left(3,1\right)=\frac{MSR-MSE}{MSR+\left(K-1\right)MSE}$$

$$ICC(3,1)=\frac{18.68-1.08}{18.68+(2-1)1.08}=0.86$$

An ICC of 0.86 was calculated, which indicated good-to-excellent interrater reliability according to established benchmarks. A Pearson correlation coefficient was also calculated between the total scores of the 2 raters, yielding a value of 0.88, further indicating a strong positive relationship and good agreement and consistency.

## Discussion

### Principal Findings

A detailed evaluation of mHealth apps for nutritional management of diabetes was conducted in this study to assess their functionality and adherence to dietary guidelines provided by the WHO, ADA, EASD, and DC. The growing reliance on mHealth apps to support patients in managing chronic conditions, such as diabetes and hypertension, has raised concerns regarding the reliability of these digital tools [22]. A total of 24 apps were selected based on predefined inclusion and exclusion criteria. Only 2 apps fully adhered to the dietary guidelines, whereas the majority demonstrated only partial compliance. The MARS assessment indicated considerable variability in overall app quality, with mean total scores ranging from 9.46 to 17.69. These findings highlight substantial shortcomings in user engagement, functionality, educational content, and personalization. The calculated ICC of 0.86 reflected good interrater reliability, and the Pearson correlation coefficient of 0.88 further indicates strong consistency between the reviewers.

### Apps Features

There was significant variation among the evaluated mHealth apps in terms of features and functionality. Most apps focused on general tracking features such as blood glucose levels, carbohydrate intake, medication adherence, and physical activity. However, many apps lacked dietary management tools, including structured meal planning, fiber intake, and personalized dietary feedback. Although these tools have the potential to support self-management, our findings indicate that many apps lack full adherence to established dietary guidelines and do not consistently deliver personalized or evidence-based feedback.

Blood glucose tracking was the most common feature, allowing users to log or sync their readings and, in some cases, visualize trends or receive insights. Apps such as mySugr and Diabetes:M (Sirma Medical Systems) provided pattern analysis based on user-entered data, which may help users adjust their diet or medication. These findings are consistent with previous studies [23], which found that diabetes apps with blood glucose tracking and data visualization are better at promoting user engagement and self-monitoring. Similarly, trend analysis and personalized insights were key features associated with high MARS scores and user satisfaction [24]. Macronutrient counting, particularly for carbohydrates, was another frequent component. Apps such as Carb Manager and Yazio offered extensive food databases and accurate meal logging, which are important for maintaining stable blood glucose levels and reducing the risk of complications such as hyperglycemia or hypoglycemia [25,26].

Furthermore, meal planning and food logging features were present in some apps to help users with suggestions for balanced meals based on their dietary preferences. However, the quality and depth of meal-planning features varied across the apps. Some showed clear nutritional information, while others lacked in this regard and provided insufficient guidance, which may negatively affect glycemic control [27]. Additionally, the cultural relevance of meal plans was limited in many apps, as most recipes or ingredients were catered toward Western dietary patterns, which may potentially reduce usefulness for diverse populations.

Reminder systems were also included in some apps, including mySugr, MyDiabetes, and Diabetes:M, to support adherence to medication or meal schedules. Push notifications and customized reminders are common features that help users adhere to schedules related to their health management [28]. However, the frequency and customization options of reminders varied across apps. Previous studies [29] have shown that the absence of reminder features can lead to low user engagement and hinder long-term adherence to the app.

Physical activity tracking was another feature that was included in most of the evaluated apps. Regular exercise is one of the most important aspects of diabetes management, as it helps improve insulin sensitivity and contributes to better blood glucose management. Regular exercise also helps in weight control, which reduces the risk of complications associated with diabetes, such as heart disease and high blood pressure. Only a few apps, such as Diabetes:M and Blood Glucose Diary for Diabetes, did not have this feature. A study showed that not all apps designed for chronic disease management include the essential components required for effective self-management, which aligns with the findings of these studies [30,31].

Additional features found in only a few apps included recipes, tracking water intake, BMI, logging blood pressure, ketones, and lipids. While these may not be essential for diabetes management, their inclusion can enhance the overall functionality of the app and support more comprehensive disease management. Overall, the presence of essential tracking tools with optional wellness features shows that current apps emphasize monitoring but often lack the integration needed for complete management of diabetes.

### Compliance With Dietary Guidelines

Maintaining blood glucose levels is important to prevent long-term complications in diabetes [32], as emphasized by major organizations including the ADA, WHO, EASD, and DC [1,9,17,18]. These guidelines consistently highlight carbohydrate control, fiber intake, glycemic monitoring, meal planning, physical activity, and patient education as the main components of diabetes management. Findings from this study showed significant variation between evaluated apps in the implementation of these recommendations, with most providing only partial adherence.

Only 2 apps, BeatO and Glycemic Index & Load App, fully met all the criteria on the compliance checklist. Several other apps, such as MyDiabetes and Glucose Buddy, provided strong carbohydrate logging but lacked features for fiber intake or detailed meal planning. Blood glucose monitoring was more commonly included, with many apps enabling users to log or sync blood glucose data, while some apps omitted this critical function. This finding is consistent with previous studies [31,33], which have shown gaps in carbohydrate and glycemic tracking that reduce the effectiveness of diabetes management apps. Inadequate carbohydrate tracking may lead to poor blood glucose management and increase the risk of complications such as diabetic neuropathy and cardiovascular disease [3]. The ability to track and analyze blood glucose levels over time improves diabetes management by providing users with information that enables them to make informed decisions about their health [33]. However, some apps, such as Yazio, Carbs Manager*,* and MyNetDiary, lacked a glucose logging feature, which may limit users’ ability to adjust their diet, medication, or exercise for effective glycemic control.

Meal planning and fiber tracking were inconsistently addressed in the evaluated apps. Carb Manager, Yazio, Glycemic Index & Load App, and MyDiabetes enabled customizable meal planning tailored to users’ medical conditions, health goals, and dietary preferences, in alignment with established dietary guidelines, while others showed limited content. Only a few apps included features that promote a fiber-rich diet, which has been shown to enhance glycemic control, reduce HbA_1c_ levels, and lower the risk of diabetes-related complications [34,35]. This result shows that the majority of apps related to diabetes management failed to incorporate core dietary recommendations from international diabetes management guidelines [36].

Most apps included features for physical activity and weight management, consistent with guideline recommendations emphasizing exercise and weight control for effective glycemic control and cardiovascular health [37,38]. However, many apps lacked these features, limiting their effectiveness in supporting users with this critical aspect of diabetes care.

Patient education, which is another important component of diabetes care, was present in fewer than half of the evaluated apps. Educational content was delivered through brief text, visuals, or links and covered basic topics such as carbohydrate counting and physical activity. Apps without educational content may limit user understanding of how to manage their condition effectively [37], while the inclusion of educational content has been associated with improved diabetes self-management and reduced risk of complications [39].

In summary, adherence to mHealth apps for diabetes management to established dietary guidelines was variable. While some apps performed well in areas such as carbohydrate control, glycemic management, meal planning, fiber intake, weight management, physical activity, and educational content, others fell short of meeting the standards set by the ADA, WHO, EASD, and DC. Similarly, a previous study [40] found that only a small percentage of diabetes apps showed full compliance with dietary guidelines through their implemented features. In this study, only 2 of the 24 apps demonstrated full adherence to the guideline compliance checklist, which highlights a persistent issue identified in both this and previous evaluations [40]. The findings emphasize the importance of incorporating features essential for diabetes management, as prescribed by dietary guidelines, to improve the effectiveness of these apps.

### MARS Evaluation

The MARS evaluation revealed variability in the quality of diabetes nutrition management apps, which showed that most apps had moderate performance and only a small portion scored highly. Overall, MARS scores among the 24 evaluated apps ranged from 9.46 to 17.69 out of a possible 20. Only 2 apps scored above 17, while one-quarter of the evaluated apps scored below 12, indicating weaknesses in user engagement, interface design, and information quality. These findings suggest that although many apps provide basic functionality, only a few have the quality required to effectively support diabetes self-management.

One of the main observations from the evaluation was that engagement was one of the weakest domains, and the apps Blood Sugar Diary for Diabetes and Diabetes App–Diabetic Diet received low scores from both authors in this category, indicating that they lacked sufficient features to keep users engaged or motivated to continue using the app. The highest score in this category was achieved by Carb Manager. Engagement is essential in encouraging users to interact with the app regularly and continue using it over time, which is important for managing chronic conditions such as diabetes. Engaging apps allow users to personalize their experience by setting goals, tracking progress, accessing interesting features, and receiving feedback, which helps in long-term adherence, increases motivation, and improves user satisfaction [41]. The absence of gamification and interactive features in many apps may further reduce sustained use. These features improve adherence and increase the frequency of blood glucose monitoring in patients with diabetes, which is consistent with the findings of this study that several apps show low performance in the engagement category [27,42].

Functionality achieved the highest mean scores among the 4 MARS domains, indicating that most apps operated reliably, were easy to navigate and performed their main tasks effectively, such as tracking carbohydrates, blood glucose, meal planning, and logging. Apps with lower scores had poorly designed user interfaces, which hindered users’ ability to navigate, locate features, and input data efficiently, potentially leading to early abandonment of the app. This finding highlights the importance of a clear and user-friendly interface as a critical factor in the overall effectiveness of mobile apps [43].

The aesthetics and information categories revealed weaknesses in most apps. Although some apps had aesthetically pleasing designs, many lacked visual appeal and consistency in design, which could lead to reduced user engagement. A clear and visually pleasing design is essential for mHealth apps to encourage frequent interaction. Poor aesthetic quality can impact user retention and satisfaction, as it can make the design too cluttered, outdated, or low quality. Such apps are likely to experience reduced user engagement over time, thereby diminishing their effectiveness as tools for diabetes management [44]. Apps often have low customer adherence due to poor user interface design, lack of aesthetic features, and poor graphics [45], which is consistent with the results of this study. The quality of information was also uneven; only a few apps provided comprehensive, evidence-based dietary content, while others included limited or outdated information. Apps that have limited visual information and lack links to information resources can result in abandonment of the app by the user [46]. Educational content within mHealth apps is crucial for improving self-management in chronic conditions through accessible knowledge for users, so they can make informed decisions about their health. This aligns with findings from a previous study [47], which showed that most diabetes self-management apps receive a moderate score on the MARS scale, particularly underperforming in the engagement and information domains, consistent with the results of this study.

In summary, the MARS analysis showed that while some mHealth apps performed well in terms of functionality and information quality, many struggled in the engagement and aesthetics domains. The limited number of high-scoring apps shows substantial room for improvement in app design, particularly in user engagement, improving visual design, and incorporating educational content into mHealth apps for diabetes management. The high interrater reliability result seen in this study further strengthens the study and its findings, which support the consistency of quality assessment.

### Strengths and Limitations

The study used established dietary guidelines from the ADA, WHO, EASD, and DC, which provided a framework for evaluating the apps. Moreover, the study used MARS, which allowed for a standardized and systematic assessment of each app.

The study also has several limitations. The sample size of 24 apps may not fully represent the entire population of mobile apps available for diabetes management. There are many apps that can offer different features specific to certain groups of people, such as children with type 1 diabetes mellitus or those managing gestational diabetes. However, those were not included in the study due to the scope and selection criteria, which focused on general nutrition management apps for diabetes. Moreover, guideline compliance was checked by binary evaluation, but it sometimes required interpretation because features were evaluated based on how closely they matched the criteria, which can vary slightly across the guidelines. Overlapping principles were prioritized where slight differences were present to maintain consistency. However, developers may structure or label features differently during app development, which poses a risk of misclassification.

Furthermore, another limitation is the subjectivity involved in scoring with the MARS tool. Several questions, particularly those in the engagement and aesthetics categories, require personal judgment, and perceptions can vary from person to person.

### Future Work

Future research should focus on evaluating the long-term impact of these apps on health outcomes, specifically investigating how sustained use of these diabetes management apps influences blood glucose management, HbA_1c_ levels, and patient adherence to lifestyle changes recommended by dietary guidelines [12,14]. Moreover, there is a need to increase the personalization of mHealth apps for the management of chronic diseases. Apps should not only provide general dietary advice but should also incorporate specific recommendations based on individual user data such as age, gender, cultural preference, and health conditions [27]. Future work could focus on integrating more adaptive learning algorithms that can offer precise and personalized feedback to users, which would improve the ability of the apps to cater to diverse populations [48]. Future studies could include patient or user feedback to strengthen research and reduce individual interpretation bias. Finally, cultural sensitivity should be considered in future app design to make diabetes management apps accessible and relevant to individuals from various cultural and ethnic backgrounds [6]. These apps can become more inclusive by incorporating localized dietary guidelines and understanding diverse cultural practices for global diabetes care.

### Conclusion

The study evaluated nutrition management mHealth apps for diabetes, their adherence to dietary guidelines, and their quality using the MARS framework.

The results show significant variations in the quality and effectiveness of the evaluated mHealth apps. Most evaluated apps demonstrated partial adherence, with only 2 apps meeting all guideline criteria, including features such as carbohydrate tracking, blood glucose monitoring, meal planning, physical activity tracking, and educational information about the condition; others failed to meet the basic standards necessary for effective diabetes management.

Moreover, in the MARS evaluation, functionality scores were generally high, but engagement, aesthetics, and information quality were limited, indicating uneven app design and content. Several lower-scoring apps exhibited poor, nonengaging designs, lacked personalization, provided limited information, and featured low-quality graphics, all of which diminish their overall effectiveness and utility as tools for disease management.

The findings of the study showed gaps between dietary guidelines and their implementation in mHealth apps, which emphasizes the need for improvement in these digital tools.

In conclusion, mHealth apps showed high potential in supporting diabetes self-management, but there is still considerable room for improvement. Developers must focus on creating apps that are not only functional but also engaging, user-friendly, high-quality, and in accordance with established dietary guidelines for diabetes. By addressing these gaps, mHealth apps can become more effective tools for diabetes management by improving patient outcomes and increasing the quality of life for individuals with this chronic condition.

## Supplementary material

10.2196/80890 Multimedia Appendix 1 Detailed overview of apps features.

10.2196/80890 Multimedia Appendix 2 Detailed overview of compliance checklist.

10.2196/80890 Multimedia Appendix 3 Questions used for the Mobile App Rating Scale (MARS) evaluation.

10.2196/80890 Multimedia Appendix 4 Mobile App Rating Scale (MARS) evaluation scores.

## Abbreviations

ADA: American Diabetes Association
CDA: Canadian Diabetes Association
DC: Diabetes Canada
EASD: European Association for the Study of Diabetes
HbA_1c_: hemoglobin A_1c_
ICC: intraclass correlation coefficient
MARS: Mobile App Rating Scale
mHealth: mobile health
MSE: mean square error
PRISMA: Preferred Reporting Items for Systematic Reviews and Meta-Analyses
WHO: World Health Organization
