# Supplementary figures and images for: Quality and Guideline Adherence of Mobile Nutrition Management Apps for Diabetes: Evaluation Study
Source: JMIR Diabetes. 2026 Jun 1;11:e80890. doi: 10.2196/80890 (PMC13225222; doi:10.2196/80890)

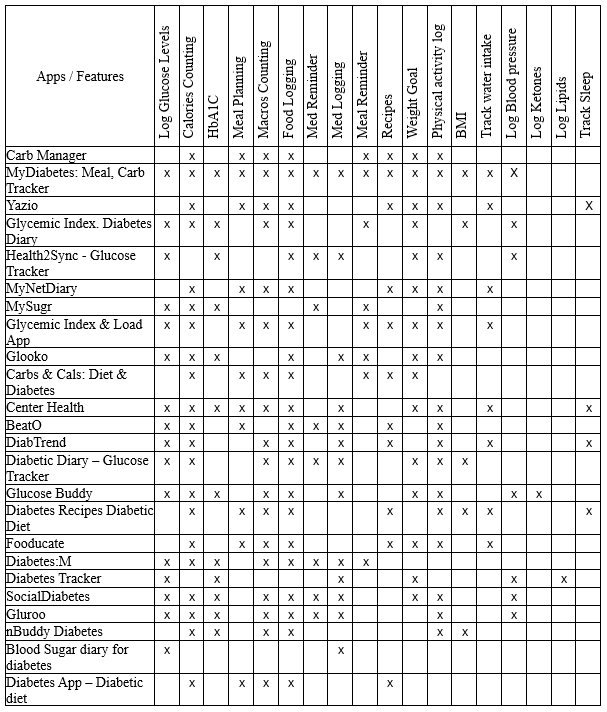

Supplement: Multimedia Appendix 1 [file diabetes-v11-e80890-s001.png]

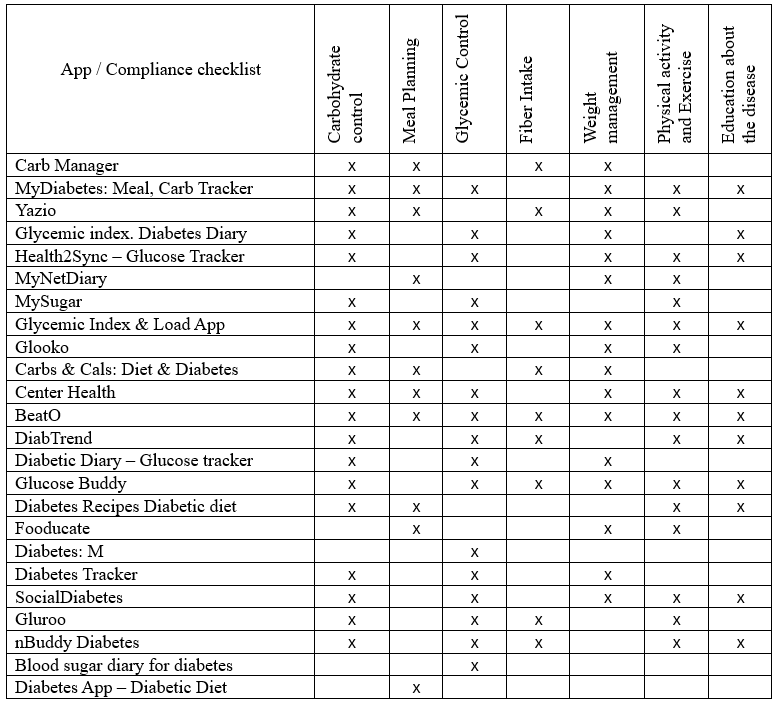

Supplement: Multimedia Appendix 2 [file diabetes-v11-e80890-s002.png]

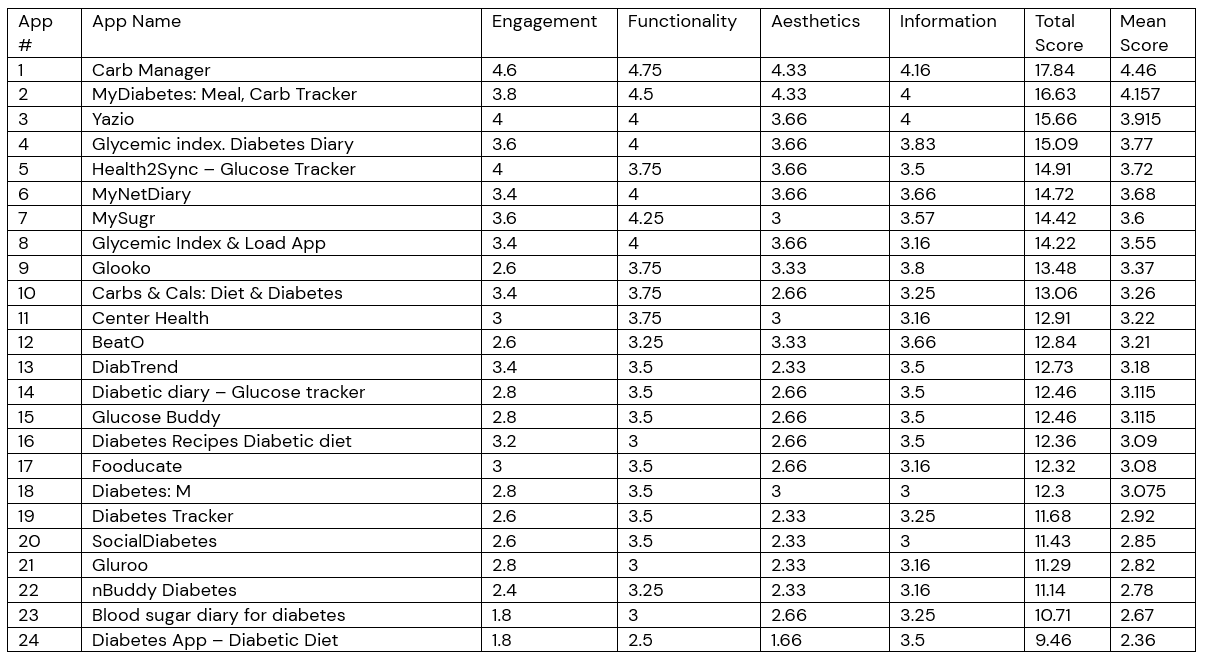

Supplement: Multimedia Appendix 4 [file diabetes-v11-e80890-s004.png]
